# Population Health Effects of Air Pollution: Fresh Evidence From China Health and Retirement Longitudinal Survey

**DOI:** 10.3389/fpubh.2021.779552

**Published:** 2021-12-23

**Authors:** Wei-Teng Shen, Xuan Yu, Shun-Bin Zhong, Hao-Ran Ge

**Affiliations:** ^1^Business School, Zhejiang Wanli University, Ningbo, China; ^2^Business School, Ningbo University, Ningbo, China; ^3^School of Information, Central University of Finance and Economics, Beijing, China

**Keywords:** air pollution, population health, physical health, mental health, hospitalization costs

## Abstract

The effects of air pollution on population health are currently a hot topic. However, few studies have examined the physical and mental health effects of air pollution jointly in China. Using data from the China Health and Retirement Longitudinal Study (CHARLS) in 2015 and 2018, this study explores how air pollution affects the physical and mental health of middle-aged and elderly residents. The empirical results highlight that air pollution can negatively affect both physical and mental health. In terms of physical health, those exposed to chronic shock are likely to suffer more adverse effects from air pollution than those exposed to acute shock. In terms of mental health, those exposed to depression suffer greater adverse effects than those exposed to episodic memory and mental cognition. Besides, heterogeneity analysis also shows that air pollution affects the mental and physical health of males more than females. Furthermore, the increase in air pollution is expected to result in huge hospitalization costs. Therefore, the Chinese government should formulate differentiated public health policies to reduce the effects of air pollution on the health of middle-aged and elderly residents.

## Introduction

This paper aims to explore whether air pollution impacts the health of China's middle-aged and elderly population, and how it affects their physical and mental health during the period of 2015–2018. Around the world, air pollution is widely recognized as a health hazard. According to the Global Burden of Disease Report 2019 (1), air pollution has caused 6.67 million deaths worldwide in 2019. As one of newly industrialized countries, China has experienced significant air pollution due to its rapid and extensive economic growth (2). As a response to increasing air pollution, the Chinese government has issued an Air Pollution Prevention and Control Action Plan in 2013. This plan includes strict monitoring of the heavy pollution sector. There have been some effects of the policy, but the situation is less than optimistic. According to the Bulletin of State of China's Ecological Environment in 2020 (3), the air quality in 40.1% of all cities at the prefecture-level and above is below the required standard. Air pollution is closely related to mortality rate (4–6). In particular, the outbreaks of infectious diseases, such as the coronavirus disease 2019 (COVID-19) can exacerbate the health effects of air pollution (7). In most parts of China, air pollution-related disabilities and deaths has exceeded 20%, and Beijing and its surrounding areas have reached about 40% (1). According to estimates by Geng et al. (8), the number of PM2.5-related deaths increased by 390,000 from 2002 to 2017, and the usage of emission control technologies for end-of-pollution prevented 870,000 deaths from air pollution.

A research hotspot has emerged around air pollution's effect on population health. Short-term exposure to air pollution may cause chronic obstructive pulmonary disease, coughing, shortness of breath, wheezing, asthma, and respiratory diseases, while long-term exposure to air pollution may cause chronic asthma, pulmonary insufficiency, cardiovascular disease, and cardiovascular death (9). There has been extensive research on whether air pollution causes the various diseases mentioned above. Particulate pollution, such as PM2.5, can damage people's lungs through their blood circulation (10, 11), increasing the risk of respiratory infections and lung cancer (4, 12). The inhalation of fine particles can result in hypercoagulability, platelet sensitization, systemic inflammation and oxidative stress. It directly or indirectly causes vascular damage, atherosclerosis and autonomic dysfunction, which could lead to cardiovascular disease (13–15). Moreover, the effects of air pollution on population health are not uniform across all groups. Children and the elderly are especially vulnerable to the adverse effects of air pollution (16–19). As a result, there have been many studies specifically focused on the effects of air pollution on the health of children (20–22) and the elderly (23, 24).

In the above research, the effects of air pollution on physical health are mainly discussed. However, air pollution can also affect mental health. For example, increasing PM2.5 concentrations can lead to negative emotional responses such as depression, nervousness, restlessness, and irritability (25–27). Two mechanisms are at work in causing the adverse effects of air pollution on mental health. On the one hand, inhaling air pollutants from the atmosphere, especially PM2.5, can cause oxidative stress and inflammatory conditions in the human body (28, 29). On the other hand, a lack of green is widely believed to be associated with mental health problems, such as depression and anxiety (30, 31), as well as cognitive impairment (32). To explain these findings, several theories have been proposed, including limitations on physical activity, biodiversity hypotheses, biogenesis theories and social stressors (33).

In summary, this study may make three marginal contributions to the literature. First of all, this study provides fresh microscopic evidence on population health effects of air pollution using data from the China Health and Retirement Longitudinal Study (CHARLS) in 2015 and 2018. CHARLS is a survey that specifically targets the health issues of middle-aged and elderly people. Comparatively with other studies that use large-scale micro-surveys to explore the health of middle-aged and elderly people (34, 35), the sample selected by CHARLS is more representative and the questionnaire design is more targeted. In addition, previous CHARLS health research mainly used cross-sectional data from a particular year (36, 37), while this study used panel data including 2015 and 2018. This allows us to control factors that do not change over time, which help us estimate how air pollution impacts health accurately.

Secondly, this study evaluates both the physical and mental health effects of air pollution. Most existing literature currently studies either the physical health or the mental health effect of air pollution without simultaneously exploring both. We find that air pollution can adversely affect physical and mental health. 10 increase of AQI will experience 0.72 increase of physical health index. An increase of 0.72% is economically significant considering average physical health index are only −0.053. The index of mental health decreases about 0.15 for every 1 increase of AQI which is equal to 23.70% of the sample mean.

Thirdly, this study enriches the measurement of physical health and mental health, and as well as analyzes the heterogeneity from different perspectives of health and gender. For physical health, existing research mainly focuses on the effect of air pollution on cardiovascular diseases or respiratory conditions. However, air pollution may also affect liver diseases, diabetes, stomach diseases, hypertension and so on. For mental health, existing research mainly concentrates on specify factors, such as depression and cognition, and cannot fully capture the effect that air pollution has on mental health. Therefore, this study constructs a comprehensive index of physical health and mental health, respectively. We find that for every one standard deviation increase in AQI, the acute shock and chronic shock will increase by 0.191 and 2.162 standard deviations, respectively. Accordingly, the episodic memory will decrease by 0.605 standard deviations, mental cognition by 0.852, and self-reported will increase by 1.032 standard deviations.

The rest of this study is organized as follows: section “Theoretical Analysis” presents the impact mechanism of air pollution on health. Section “Data and Methodology” describes data source, variables selection and methodology. The empirical results are presented and discussed in the section “Empirical Results”. Section “Conclusions and implications” concludes the paper.

## Theoretical Analysis

Air pollution can affect health in many ways. It can have a negative effect on physical health. Firstly, air pollution can directly cause damage to human cells, tissues and organs. Air pollutants can directly lead to the disruption of the airway epithelial barrier and cell signaling pathways (38), injury from free radical peroxidation (39), imbalanced intracellular calcium homeostasis (39), Inflammatory injury (40), cellular immune disorders (41), epigenetic modification (42), and autophagy (43), thereby causing chronic respiratory diseases (39, 44). Air pollution can also cause cardiovascular disease. Pollutants can cause epigenetic changes with downstream effects on the cardiovascular system. They also activate inflammatory cells in the lungs, including macrophages, leading to increased systemic inflammation and oxidative stress, thereby cardiac dysfunction (45). In addition, exposure to pollution can cause sleep quality decline or insomnia caused by shortness of breath, increased heart rate, upper respiratory tract irritation, thereby cause other diseases (46). Secondly, aerosol particles can scatter and absorb surface solar radiation directly. As cloud condensation nuclei, aerosols can indirectly affect the solar radiation on the ground by changing the number of cloudiness (47, 48). The lack of sunlight will not be conducive to the formation of vitamin D, thereby increasing the risk of chronic diseases and cancer (49). Thirdly, people might reduce outdoor activity time during a severe air pollution episode (50), thereby insufficient exercise. Lack of exercise may lead to health problems such as reduced human organ function, slowed blood circulation, slowed metabolism, weakened immunity, and other health problems. Finally, air pollution will increase the probability of workers getting sick, reduce working hours, work efficiency (51), thereby a decline in workers' income.

Air pollution can also adversely affect people's mental health. First of all, the fine particles that cause air pollution may increase the body's oxidative stress and systemic inflammation. These changes can severely destroy the cytokine signals that regulate brain function, manifested as depression (52), anxiety (53) and cognitive dysfunction (54, 55). In addition, air pollution can induce a variety of physical health diseases, such as cardiovascular, respiratory, and obstructive lung diseases. The patients who are suffering from illness will have a negative psychological status. This is harmful to mental health and may even increase the risk of suicide (56). Finally, to reduce the potential risk of exposure to air pollution, people may increase the time spent indoors. Staying indoors for a long time can increase people's feelings of anxiety and loneliness which is harmful to mental health (57). Air pollution can also have an indirect impact on mental health. Air pollution may cause macroeconomic fluctuations by reducing enterprise performance and hindering foreign direct investment (58). The deterioration of the macro economy will cause workers to worry about future job loss and income reduction, which will adversely affect mental health (59).

## Data and Methodology

### Data Source

The dataset of individual characteristic variables has been collected from the China Health and Retirement Longitudinal Study (CHARLS) in 2015 and 2018. So far, CHARLS has conducted the first, second, third, and fourth rounds of investigations in 2011, 2013, 2015, and 2018, respectively. The reason why we excluded 2011 and 2013 from the analysis is the items on the mental health part of the questionnaires in 2011 and 2013 are incomplete, and the questionnaires in 2015 and 2018 are more complete and unify than 2011 and 2013. The CHARLS is organized and carried out by the National Development Research Institute of Peking University, and it is a biannual and nationally representative survey of middle-aged and elderly population aged 45 and above. The CHARLS national baseline was carried out in 2011 and then tracked every 2–3 years. The data covers approximately 17,708 residents in 150 county-level units and 450 village-level units in China. Detailed descriptions of individual sampling methods are provided in the user manual compiled by Zhao et al. (60). There is a wide range of information provided in CHARLS, including health status and function, health care and insurance, cognition and depression, and other individual basic information. Therefore, CHARLS is a representative data to study population health and its influencing factors among middle-aged and elderly people.

### Variable Selection

#### Dependent Variable

The dependent variable is population health, which is measured from two dimensions: physical health and mental health. In terms of physical health, it can be measured by acute and chronic shock according to the disease characteristics. In terms of mental health, it is evaluated by three aspects: episodic memory, mental cognition and self-reported depression. Due to the fact that different indicators have different dimensions, this study standardizes the secondary indicators and then carries out a simple arithmetic average method to obtain the sum of physical health and mental health, respectively. Specially, Table 1 presents the constructed indicators for measuring physical health and mental health.

**Table 1:** Indicators for measuring physical and mental health.

**Primary indicators**	**Secondary indicators**	**Definition of indicator**	**Indicator attribute**
Physical health	Acute shock	One point is scored for each of the three diseases of stroke, heart disease, and cancer. The range of values is [0, 3].	The higher value of this indicator, the stronger of acute shock and the worser of physical health.
	Chronic shock	One point is scored for each of hypertension, diabetes, kidney, chronic lung, asthma, dyslipidemia, liver disease, stomach disease, and arthritis. The range of values is [0, 9].	The higher value of this indicator, the stronger of chronic shock and the worser of physical health.
Mental health	Episodic memory	The memory includes short-term memory and delayed memory. There are 20 questions to answer, and each correct answer is worth one point. The range of values is [0, 20].	The higher value of this indicator, the stronger of the ability of episodic memory and the healthier of mental health.
	Mental cognition	Answer questions about dates, seasons, drawings, calculations, etc., and mark one point for each correct answer. The range of values is [0, 12].	The higher value of this indicator, the stronger of the ability of mental cognition and the healthier of mental health.
	Self-reported depression	It contains 10 questions about feelings and behaviors in the last week. Respondents choose from four options that indicate the frequency and add the representative scores of the options to obtain a self-reported depression score. The range of values is [0, 40].	The higher value of this indicator, the higher of the self-reported depression and the worse of mental health.

#### Independent Variable

The core independent variable of this study is the air pollution. According to the current literature on air pollution in China, the main measurement indicators of air pollution are the air quality index (AQI), the air pollution index (API), PM2.5, SO2, CO, NO2 and O3 (4, 20, 46, 61–63). Among them, AQI has recently been widely accepted as the most reliable and representative indicator for measuring air pollution. AQI is calculated as a composite indicator of PM2.5, PM10, SO2, CO, NO2, and O3, ranging from 0 to 500. The higher the AQI, the worse the air pollution. Hence, this study collects the daily AQI of prefecture-level cities in 2015 and 2018 from the China National Environmental Monitoring Center, and then calculates the annual average of AQI of each city in 2015 and 2018. In addition, this study also uses two common single indicators of PM2.5 and PM10 respectively in the robustness check.

#### Control Variable

The control variables include individual-level and city-level variables. Refer to previous related studies of Gu et al. (64), Hu et al. (65), and Giaccherini et al. (66), this study controls for the individual characteristics including education level, age, gender, marital status, sleep status, whether they have medical insurance, and whether they are accompanied by children. Meanwhile, this study also controls for the urban characteristics including waste treatment rate, sewage treatment rate, population density, medical level, and economic development level.

#### Variable Description

Table 2 shows the definition and descriptive statistics of each variable in this study. As Table 2 shows, the values of physical and mental health range from−1.005 to 5.205 and−4.680 to 4.262, respectively, indicating that the health status varies greatly among individuals. The average value of AQI is 76.590, suggesting that air pollution in China is severe in 2015 and 2018. In terms of individual characteristics, the average values of education level and gender are 6.650 and 0.480, which means that the sample data used in this study has good representativeness. In terms of urban characteristics, the average centralized sewage treatment and harmless garbage disposal rates reach 90.903% and 95.729% respectively. It implies that the impacts of environmental pollution on population health may also an important factor in the sample data.

**Table 2:** Descriptive statistics.

**Variable**	**Definition**	**Obs**	**Mean**	**SD**	**Min**	**Max**
Phyheal	The higher the value of Phyheal, the worse the physical health	39,492	−0.053	1.117	−1.005	5.205
Menheal	The higher the value of Menheal, the better the mental health	17,250	0.638	1.990	−4.680	4.262
AQI	AQI index (The larger the value, the more serious the air pollution)	36,129	76.590	22.211	33.971	138.504
Education	Education level (The higher the value, the higher the level of education)	39,634	6.650	4.439	1.000	12.000
Age	Age of the resident	38,712	61.105	10.079	45.000	87.000
Gender	1= male, 0 = female	39,650	0.480	0.500	0.000	1.000
Married	Married=1, unmarried=0	39,617	0.857	0.350	0.000	1.000
Lwchi	Accompanied by children, 1 = yes, 0 = no	20,979	0.405	0.491	0.000	1.000
Sleep	Sleep quality (The larger the value, the worse the sleep quality)	36,239	2.080	1.217	1.000	4.000
Medins	Whether the respondent enrollment in social basic medical insurance, 1 = yes, 0 = no	19,540	0.970	0.171	0.000	1.000
sewpro	Centralized sewage treatment rate (%)	33,455	90.903	6.994	64.430	100.000
gartre	Harmless garbage disposal rate (%)	34,067	95.729	9.654	40.700	100.000
bedper	Number of hospital beds per 10,000 people	35,677	47.010	16.879	21.895	98.333
lndensity	Logarithm of population density	35,756	5.933	0.905	2.281	7.113
lnpergdp	Logarithm of GDP per capita	35,756	10.703	0.652	9.382	12.431

### Methodology

According to Grossman's health demand theory (67), the health capital stock could be influenced by age, income, environmental pollution level, lifestyle, education, and other factors. Based on the health demand theory and the settings of Chen et al. (20), Liao et al. (4), Heyes and Zhu (46), we constructed the following model:


$$\begin{array}{l} Health_{ict=}\alpha_{0}+\alpha_{1}Airpollution_{ict}+\overset{12}{\underset{v=2}{\sum }}\alpha_{\;v}Contronl_{v}+\gamma_{c}+\lambda_{t}\\ \;+\varepsilon_{ict} \end{array}$$

where *Health*_*ict*_ is the health level of individual *i* living in city *c* in year *t*. In this study, health is further divided into physical health and mental health, respectively. *Airpollution*_*ict*_ is the air pollution level of individual *i* living in city *c* in year *t*. *Contronl*_*v*_ represents the control variables, including individual and urban characteristics. γ_*c*_ is the city fixed effects. λ_*t*_ is the year fixed effects. The city and year fixed effects are introduced to control the unobservable factors changing with city and time. ε_*ict*_ is the error term. α_0_, α_1_, …α_12_ are the parameters to be estimated.

## Empirical Results

### Baseline Regression Results

The results of the baseline regression are reported in Table 3. Columns (1) and (2) show the estimated results of air pollution on residents' physical health and mental health, respectively. As shown in column (1), the coefficient of AQI is positive and significant at the 1% level, implying that increasing air pollution will be detrimental to physical health. The estimated coefficient value of AQI is 0.0723, suggesting that every 10 units increase of AQI will experience 0.72 increase of index of physical health. It's worth noting that an increase of 0.72 is also economically significant considering average physical health index are only −0.053. In addition, as shown in column (2), the coefficient of AQI is negative and significant at the 1% level, indicating that increasing air pollution is also harmful to mental health. Specifically, the estimated coefficient −0.1512 means that the index of mental health decreases about 0.15 for every 1 increase of AQI. This is equal to 23.70% of the sample mean, suggesting that air pollution contributes a large negative effect to residents' mental health. Existing studies have found that air pollution can increase the risk of diseases such as dementia (68), asthma (69), depression (26), and lung cancer (10). The results of these studies provide some support for the above findings.

**Table 3:** The effects of air pollution on physical and mental health.

	**(1)**	**(2)**
	**Phyheal**	**Menheal**
AQI	0.0723^***^ (0.0019)	−0.1512^***^ (0.0029)
Education	0.0022 (0.0101)	0.4595^***^ (0.0160)
Age	0.0123^***^ (0.0019)	−0.0329^***^ (0.0030)
Gender	0.0110 (0.0443)	0.0325 (0.0494)
Married	0.0046 (0.0370)	0.4249^***^ (0.0592)
Lwchi	-0.0190 (0.0280)	0.0407 (0.0396)
Sleep	0.1293^***^ (0.0158)	−0.5193^***^ (0.0240)
Medins	0.2109^***^ (0.0712)	0.5155^**^ (0.1906)
sewpro	-0.0349^***^ (0.0017)	0.1139^***^ (0.0021)
gartre	0.0841^***^ (0.0020)	−0.1610^***^ (0.0035)
bedper	0.0033^***^ (0.0001)	−0.0165^***^ (0.0004)
lndensity	0.2754^***^ (0.0117)	0.0801^***^ (0.0135)
lnpergdp	0.2481^***^ (0.0115)	−0.4676^***^ (0.0225)
City fixed effects	Yes	Yes
Year fixed effects	Yes	Yes
Constant	-15.9956^***^ (0.3278)	22.2470^***^ (0.5363)
Observations	7624	6098
Adjusted *R*^2^	0.038	0.444

*Standard errors in parentheses, clustered at the Provincial level*.

^*^, ^**^, and ^***^ indicate significance at the levels of 10, 5, and 1%, respectively.

### Robustness Checks

#### Using Other Dependent Variables

Although multiple options are integrated to construct population health, some key health issues may still be overlooked. For robustness checks, we use the numbers of hospitalization to measure physical health. Here we actually assume that the more hospitalizations, the worse the physical health. As shown in column (1) of Table 4, the regression coefficient of AQI is positive and significant at the 1% level. The estimated results indicate that increasing air pollution will result in more hospitalizations, and more hospitalizations reflect poorer physical health. As a consequence, the conclusion in the baseline that air pollution is bad for population health still remains valid.

**Table 4:** Results after changing the measurement of the dependent variable.

	**(1)**	**(2)**
	**Hosptime**	**Menheal2**
AQI	0.0492^***^ (0.0009)	0.0099^***^ (0.0003)
Education	0.0004 (0.0043)	0.0015 (0.0012)
Age	0.0107^***^ (0.0008)	0.0002 (0.0003)
Gender	0.0231 (0.0148)	−0.0126^**^ (0.0055)
Married	-0.0125 (0.0268)	−0.0159^*^ (0.0091)
Lwchi	0.0021 (0.0122)	−0.0072 (0.0080)
Sleep	0.0423^***^ (0.0039)	0.0164^***^ (0.0018)
Medins	0.1513^***^ (0.0317)	−0.0069 (0.0225)
sewpro	-0.0390^***^ (0.0009)	−0.0052^***^ (0.0003)
gartre	0.0665^***^ (0.0011)	0.0119^***^ (0.0004)
bedper	0.0048^***^ (0.0001)	−0.0010^***^ (0.0000)
lndensity	0.0643^***^ (0.0055)	−0.0066^***^ (0.0023)
lnpergdp	0.1886^***^ (0.0058)	0.1119^***^ (0.0021)
City fixed effects	Yes	Yes
Year fixed effects	Yes	Yes
Constant	-9.6388^***^ (0.1511)	−2.5107^***^ (0.0454)
Observations	7628	7624
Adjusted *R*^2^	0.053	0.012

*Standard errors in parentheses, clustered at the Provincial level*.

^*^, ^**^, and ^***^ indicate significance at the levels of 10, 5, and 1%, respectively.

In the CHARLS questionnaire, there is a question entitled, “Are you currently using these methods to treat mental, psychological, or emotional disorders?” This question is for respondents with a mental, psychological, or emotional illness. The options for this question include “1. Receiving psychiatric or psychological treatment; 2. Taking anti-depressants; 3. Taking tranquilizers or sleeping pills; 4. Other treatments, please specify; 5. None of the above”. We set a mental health variable based on the options of the question, and the value is 1 when no corresponding treatment measures are taken, 2 when one of the treatment measures is taken, and so on. The value of this new mental health variable ranges from 1 to 5. The larger the value, the worse the mental health. In Table 4, column (2) reports the results with new mental health variable as the dependent variable. The results show that the coefficient of AQI is positive and significant at the 1% level, indicating that increasing air pollution will reduce mental health level. This finding is consistent with the conclusion obtained from Table 3.

#### Using Other Independent Variables

As Cheng et al. (70) and Yang et al. (71) have shown, PM2.5 and PM10 are the main pollutants causing air pollution Chinese cities. Hence, we also take them as independent variables for robustness checks. The estimation results are reported in columns (1)–(4) of Table 5. As shown in columns (1) and (3), the regression coefficients of PM2.5 and PM10 are all positive and significant at the 1% level. The results indicate that an increase in PM2.5 and PM10 concentrations will adversely affect residents' physical health. Meanwhile, the regression coefficients of PM2.5 and PM10 in columns (2) and (4) are all negative and significant at the 1% level, implying that an increase in PM2.5 and PM10 concentrations will also be detrimental to residents' mental health. These results further verify the reliability of the conclusion that the increase of air pollution will pose a threat to both physical health and mental health.

**Table 5:** Results of PM2.5 and PM10 as the independent variables.

	**(1)**	**(2)**	**(3)**	**(4)**
	**Phyheal**	**Menheal**	**Phyheal**	**Menheal**
PM2.5	0.0730^***^ (0.0019)	−0.1527^***^ (0.0029)		
PM10			0.1259^***^ (0.0033)	−0.2634^***^ (0.0050)
Education	0.0022 (0.0101)	0.4595^***^ (0.0160)	0.0022 (0.0101)	0.4595^***^ (0.0160)
Age	0.0123^***^ (0.0019)	−0.0329^***^ (0.0030)	0.0123^***^ (0.0019)	−0.0329^***^ (0.0030)
Gender	0.0110 (0.0443)	0.0325 (0.0494)	0.0110 (0.0443)	0.0325 (0.0494)
Married	0.0046 (0.0370)	0.4249^***^ (0.0592)	0.0046 (0.0370)	0.4249^***^ (0.0592)
Lwchi	-0.0190 (0.0280)	0.0407 (0.0396)	-0.0190 (0.0280)	0.0407 (0.0396)
Sleep	0.1293^***^ (0.0158)	−0.5193^***^ (0.0240)	0.1293^***^ (0.0158)	−0.5193^***^ (0.0240)
Medins	0.2109^***^ (0.0712)	0.5155^**^ (0.1906)	0.2109^***^ (0.0712)	0.5155^**^ (0.1906)
sewpro	−0.0104^***^ (0.0011)	0.0626^***^ (0.0014)	−0.1505^***^ (0.0046)	0.3557^***^ (0.0065)
gartre	0.0681^***^ (0.0017)	−0.1275^***^ (0.0029)	0.1984^***^ (0.0050)	−0.4001^***^ (0.0080)
bedper	−0.0173^***^ (0.0005)	0.0267^***^ (0.0011)	0.0514^***^ (0.0013)	−0.1171^***^ (0.0018)
lndensity	0.1477^***^ (0.0111)	0.3471^***^ (0.0159)	0.9225^***^ (0.0236)	−1.2736^***^ (0.0249)
lnpergdp	0.5064^***^ (0.0181)	−1.0078^***^ (0.0326)	0.2144^***^ (0.0107)	−0.3971^***^ (0.0212)
City fixed effects	Yes	Yes	Yes	Yes
Year fixed effects	Yes	Yes	Yes	Yes
Constant	−15.7287^***^ (0.3217)	21.6888^***^ (0.5266)	−27.4986^***^ (0.6085)	46.3083^***^ (0.9733)
Observations	7624	6098	7624	6098
Adjusted *R*^2^	0.038	0.444	0.038	0.444

Standard errors in parentheses, clustered at the Provincial level. ^x0002A;^, ^**^, and ^***^ indicate significance at the levels of 10, 5, and 1%, respectively.

### Heterogeneous Effects of Air Pollution

#### Heterogeneity of Subgroups

The impact of air pollution on population health may vary among different types of physical health or mental health. Thus, this study investigates the heterogeneous effects of air pollution on physical health based on acute and chronic shocks, and analyzes how air pollution impacts mental health based on episodic memory, mental cognition, and self-reported depression. The estimation results are shown in Table 6.

**Table 6:** Results of heterogeneity test based on the secondary indicators of physical and mental health.

	**Physical health**		**Mental health**		
	**(1)**	**(2)**	**(3)**	**(4)**	**(5)**
	**Acute shock**	**Chronic shock**	**Episodic memory**	**mental cognition**	**Self-reported depression**
AQI	0.0021^***^ (0.0004)	0.0626^***^ (0.0012)	-0.0994^***^ (0.0026)	−0.1281^***^ (0.0062)	0.3004^***^ (0.0080)
Education	0.0012 (0.0023)	0.0009 (0.0069)	0.4950^***^ (0.0152)	0.5900^***^ (0.0312)	-0.4699^***^ (0.0502)
Age	0.0036^***^ (0.0005)	0.0018^**^ (0.0008)	-0.0587^***^ (0.0028)	−0.0270^***^ (0.0045)	0.0073 (0.0061)
Gender	-0.0102 (0.0091)	0.0316 (0.0239)	-0.2859^***^ (0.0514)	0.3001^***^ (0.0905)	-0.5391^***^ (0.1152)
Married	0.0045 (0.0089)	−0.0111 (0.0235)	0.3257^***^ (0.0648)	0.3611^***^ (0.0805)	-0.8342^***^ (0.1639)
Lwchi	0.0019 (0.0069)	−0.0293^*^ (0.0151)	0.0404 (0.0403)	−0.0343 (0.0648)	-0.2138^*^ (0.1154)
Sleep	0.0179^***^ (0.0037)	0.0669^***^ (0.0076)	−0.1137^***^ (0.0213)	−0.1136^***^ (0.0263)	2.8608^***^ (0.0696)
Medins	0.0576^***^ (0.0199)	0.0420 (0.0500)	0.3294^**^ (0.1338)	0.8226^***^ (0.1865)	−0.4118 (0.4121)
sewpro	0.0029^***^ (0.0004)	−0.0412^***^ (0.0010)	0.1138^***^ (0.0021)	0.0812^***^ (0.0048)	−0.2222^***^ (0.0058)
gartre	0.0010^**^ (0.0004)	0.0777^***^ (0.0013)	−0.1058^***^ (0.0032)	−0.1431^***^ (0.0071)	0.2952^***^ (0.0097)
bedper	0.0014^***^ (0.0000)	−0.0023^***^ (0.0001)	−0.0039^***^ (0.0002)	−0.0282^***^ (0.0005)	0.0620^***^ (0.0009)
lndensity	0.0323^***^ (0.0026)	0.0990^***^ (0.0066)	−0.3591^***^ (0.0190)	0.0059 (0.0280)	−1.5616^***^ (0.0459)
lnpergdp	−0.0528^***^ (0.0024)	0.4278^***^ (0.0075)	−0.5363^***^ (0.0177)	0.0446 (0.0435)	0.2657^***^ (0.0549)
City fixed effects	Yes	Yes	Yes	Yes	Yes
Year fixed effects	Yes	Yes	Yes	Yes	Yes
Constant	−0.4158^***^ (0.0733)	−13.1453^***^ (0.2229)	19.9940^***^ (0.5204)	21.6376^***^ (1.2856)	−10.8306^***^ (1.6038)
Observations	7624	7624	7629	6656	6841
Adjusted *R*^2^	0.032	0.023	0.368	0.321	0.402

Standard errors in parentheses, clustered at the Provincial level. ^*^, ^**^, and ^***^ indicate significance at the levels of 10, 5, and 1%, respectively.

In terms of physical health, columns (1) and (2) of Table 6 take acute shock and chronic shock as the dependent variables. The coefficients of AQI are both positive and significant at the 1% level, indicating that air pollution will increase the incidence of acute and chronic diseases, but it is more likely to cause the occurrence of chronic diseases than acute diseases. When taking episodic memory and mental cognition as the dependent variables in columns (3) and (4), the result show that the coefficients of AQI are both negative and significant at the 1% level. It suggests that Memory and cognitive ability of middle-aged and elderly people will be impaired by air pollution. Moreover, the coefficients of AQI in column (5) is also negative and significant at the 1% level, indicating that depression will rise among the middle-aged and the elderly due to an increase in air pollution. Therefore, air pollution has a systemic effect on the health of middle-aged and elderly people.

Furthermore, according to Table 2, a standard deviation of 22.211, 0.244, 0.643, 3.652, 3.339, and 6.463 are found in the AQI, acute shock, chronic shock, episodic memory, psychological cognition, and self-reported depression, respectively. Thus, the estimation results in Table 6 can be expressed as for every one standard deviation increase in AQI, the acute shock and chronic shock will increase by 0.191 and 2.162 standard deviations, respectively. Accordingly, the episodic memory will decrease by 0.605 standard deviations, mental cognition by 0.852, and self-reported depression will increase by 1.032 standard deviations.

#### Heterogeneity of Gender

The effects of air pollution on population health may differ among males and females due to differences in physical structures and lifestyles. Thus, we test the heterogeneous effects of air pollution on physical and mental health between men and women, and present the results in Table 7. In both male and female samples in columns (1)–(4), the direction of the effects of air pollution on physical health and mental health is similar, but the degree of the effects differs. Moreover, male samples have larger coefficients of AQI than female samples, and the AQI coefficient difference tests for male and female samples are statistically significant. Therefore, we can conclude that air pollution adversely affects population health of males more than females. A similar result is obtained by Gu et al. (64).

**Table 7:** Results of heterogeneity test based on gender.

	**Physical health**		**Mental health**	
	**(1)**	**(2)**	**(3)**	**(4)**
	**Male**	**Female**	**Male**	**Female**
AQI	0.1381^***^ (0.0038)	0.0439^***^ (0.0022)	−0.1678^***^ (0.0057)	−0.1511^***^ (0.0029)
Education	0.0063 (0.0128)	0.0023 (0.0146)	0.3802^***^ (0.0228)	0.5166^***^ (0.0168)
Age	0.0099^***^ (0.0026)	0.0146^***^ (0.0022)	−0.0328^***^ (0.0038)	−0.0329^***^ (0.0040)
Gender	0.0000 (0.0000)	0.0000 (0.0000)	0.0000 (0.0000)	0.0000 (0.0000)
Married	0.0148 (0.0531)	0.0069 (0.0529)	0.3983^***^ (0.1059)	0.4225^***^ (0.0745)
Lwchi	−0.0151 (0.0403)	−0.0281 (0.0440)	−0.0235 (0.0630)	0.1015 (0.0717)
Sleep	0.1368^***^ (0.0205)	0.1221^***^ (0.0195)	−0.4992^***^ (0.0280)	−0.5315^***^ (0.0260)
Medins	0.0596 (0.1401)	0.3070^**^ (0.1198)	0.4794^*^ (0.2449)	0.5666^**^ (0.2307)
sewpro	−0.0919^***^ (0.0030)	−0.0096^***^ (0.0019)	0.1382^***^ (0.0036)	0.0932^***^ (0.0030)
gartre	0.1594^***^ (0.0048)	0.0509^***^ (0.0022)	−0.1856^***^ (0.0067)	−0.1575^***^ (0.0026)
bedper	0.0014^***^ (0.0002)	0.0016^***^ (0.0002)	−0.0224^***^ (0.0005)	−0.0110^***^ (0.0007)
lndensity	0.5508^***^ (0.0119)	0.0459^**^ (0.0197)	0.1796^***^ (0.0231)	0.0960^***^ (0.0279)
lnpergdp	0.9754^***^ (0.0299)	−0.0583^***^ (0.0092)	−0.6070^***^ (0.0487)	−0.4303^***^ (0.0196)
City fixed effects	Yes	Yes	Yes	Yes
Year fixed effects	Yes	Yes	Yes	Yes
Constant	−32.0890^***^ (0.6985)	−8.4218^***^ (0.3669)	25.2380^***^ (1.1593)	22.7466^***^ (0.7032)
Observations	3629	3995	3064	3034
Adjusted *R*^2^	0.029	0.044	0.353	0.499
Chi2	465.88^***^		8.45^***^	

Standard errors in parentheses, clustered at the Provincial level. *, **, and *** indicate significance at the levels of 10%, 5%, and 1%, respectively. Chi2 is the statistics obtained by the difference test between groups for AQI, and Chi2_p is the corresponding P-value.

Why does air pollution affect men's health more than women? On the one hand, Males and females may be affected by air pollution differently because males spend more time outdoors than females, so they are exposed to air pollution for a longer period of time. On the other hand, there are two major tissues of the human central nervous system: gray matter and white matter. Mathematical ability is often reflected in gray matter, while language expression ability is reflected in white matter (72). Furthermore, there are more activated gray cells in the brains of men than females in the intelligence test (73). In this case, those exposed to air pollution would also have diminished mathematical ability, but more pronounced for males than females. In this case, it makes sense that air pollution has a greater impact on mental health in men since mental cognition is primarily based on mathematical ability.

### Further Analysis: Hospital Costs of Air Pollution

Air pollution adversely affects physical and mental health, as demonstrated above. The question that remains then is how much are hospital costs caused by air pollution. Identifying the answer to this question is central to evaluating air pollution control policies' welfare effects. As show in Table 8, air pollution, represented by AQI, PM2.5, and PM10, can significantly improve the hospitalization costs of middle-aged and elderly people. That is, air pollution increases the medical costs of middle-aged and elderly people. This conclusion is consistent with the finding obtained by Zeng et al. (74) using provincial-level data. In terms of AQI in columns (1), hospitalization costs increase by about 19.06% for every 1 unit increase in AQI. In terms of PM2.5 in columns (2), for every 1 μg/m^3^ increase in PM2.5, hospitalization costs will increase by about 19.26%. In terms of PM10 in columns (3), for every 1 μg/m^3^ increase in PM10, hospitalization costs will also increase by about 33.22%. As a result, it can be concluded that air pollution will result in huge hospital expenditures.

**Table 8:** The effects of air pollution on hospital costs.

	**(1)**	**(2)**	**(3)**
	**lnHospcost**	**lnHospcost**	**lnHospcost**
AQI	0.1906^***^ (0.0037)		
PM2.5		0.1926^***^ (0.0037)	
PM10			0.3322^***^ (0.0064)
Education	0.0065 (0.0153)	0.0065 (0.0153)	0.0065 (0.0153)
Age	0.0315^***^ (0.0041)	0.0315^***^ (0.0041)	0.0315^***^ (0.0041)
Gender	0.0564 (0.0575)	0.0564 (0.0575)	0.0564 (0.0575)
Married	−0.0144 (0.0862)	−0.0144 (0.0862)	−0.0144 (0.0862)
Lwchi	0.0433 (0.0433)	0.0433 (0.0433)	0.0433 (0.0433)
Sleep	0.1231^***^ (0.0151)	0.1231^***^ (0.0151)	0.1231^***^ (0.0151)
Medins	0.3948^***^ (0.1271)	0.3948^***^ (0.1271)	0.3948^***^ (0.1271)
sewpro	−0.1540^***^ (0.0035)	−0.0893^***^ (0.0023)	−0.4590^***^ (0.0092)
gartre	0.2547^***^ (0.0043)	0.2124^***^ (0.0036)	0.5562^***^ (0.0101)
bedper	0.0181^***^ (0.0003)	−0.0363^***^ (0.0010)	0.1449^***^ (0.0026)
lndensity	0.2415^***^ (0.0196)	−0.0953^***^ (0.0196)	1.9487^***^ (0.0409)
lnpergdp	0.9079^***^ (0.0250)	1.5892^***^ (0.0376)	0.8190^***^ (0.0234)
City fixed effects	Yes	Yes	Yes
Year fixed effects	Yes	Yes	Yes
Constant	−38.3107^***^ (0.6816)	−37.6067^***^ (0.6698)	−68.6556^***^ (1.2243)
Observations	6770	6770	6770
Adjusted *R*^2^	0.042	0.042	0.042

Standard errors in parentheses, clustered at the Provincial level. ^*^, ^**^, and ^***^ indicate significance at the levels of 10, 5, and 1%, respectively.

This estimation result is quite different from the study of Liao et al. (4). Their study showed that hospitalization costs increase by about 2% for every 10 μg/m^3^ increase in PM2.5 concentration. This difference can be attributed to the fact that Liao et al. used data from China Family Panel Studies (CFPS), which includes all family members in the sample households. The CHARL used in this study mainly targets the middle-aged and elderly people in the sample households, among which about 36% of the population are over 65 years old. Elderly people have poor physical fitness, are sensitive to environmental changes, and are more susceptible to air pollution, which can lead to diseases related to aging (75), thereby spend more on hospitalization costs than young people (9).

## Conclusions and Implications

Based on the CHARLS data in 2015 and 2018, this study applies a panel fixed effects model to empirically test the population health effects of air pollution among middle-aged and elderly residents. First, we divide population health into physical health and mental health, and then test the health effects of air pollution on them. The results indicate that air pollution is harmful to both physical and mental health. Each 10 increase of AQI will cause the physical health score to drop by 0.72, and each 1 increase of AQI will contribute a decrease of about 0.15 to mental health, which is equal to 23.70% of the sample mean.

Second, heterogeneity tests are performed on the population health effects of air pollution based on both health categories and gender, respectively. In terms of physical health, air pollution can increase acute shock and chronic shock, but it has a greater impact on chronic shock than acute shock. In terms of mental health, air pollution can impair memory and cognitive abilities and exacerbate depression. In addition, the effects of air pollution on physical and mental health differ significantly between males and females. Air pollution has a more detrimental effect on men than on women.

Third, the hospital costs of air pollution are examined in this study. The estimation results using AQI, PM2.5, and PM10 are all consistent with the fact that air pollution will lead to a huge increase in hospitalization costs.

Important policy implications can be drawn from this study as well. To begin with, prior to formulating public health policies, it is important to take into account both physical and mental health effects of air pollution. The government can set up health consultation centers for middle-aged and elderly people in the community so that they can identify the physical and mental risks that middle-aged and elderly people face. Moreover, it is also important to develop differentiated environmental public health policies based on the differing effects of air pollution on acute shock, chronic shock, memory ability, cognitive ability, and depression. The government should determine the level of financial investment according to the level of adverse health effects caused by air pollution. Furthermore, government health departments should develop guidelines that differentiate the effects of air pollution on males and females.

However, there are still some shortcomings in this study. In addition to directly harming the health of middle-aged and elderly people by causing some diseases, air pollution may indirectly harm them by aggravating diseases they already have. It is important to differentiate between direct and indirect effects in order to better manage pollution-related adverse health effects. Nonetheless, this study is unable to test the direct and indirect effects due to non-availability of data. With the possible acquisition of tracking data for individual disease monitoring, it may be possible in the future to make a detailed analysis of the direct and indirect effects of air pollution on health. In addition, due to the lack of data, this study has not been able to discuss related issues caused by COVID-19 pandemic. COVID-19 pandemic may result in a large number of people losing their jobs (76, 77), thereby lowering their living standards, which may exacerbate air pollution's adverse effects on health. In comparison with studies on air pollution's impact on health, there are relatively few studies on the factors that impact air pollution, such as environmental innovation, environmental taxes (78), and other factors impact pollution levels. We will further expand on the basis of this study and explore how to reduce air pollution in the future.

## Data Availability Statement

The raw data supporting the conclusions of this article will be made available by the authors, without undue reservation.

## Author Contributions

W-TS: conceptualization, analyzing, and writing—original draft. XY: writing and reviewing. S-BZ: writing, editing, and supervising. H-RG: data preparation and software. All authors contributed to the article and approved the submitted version.

## Funding

This research was partly supported by the Soft Science Research Plan Project of Zhejiang Province (grant number 2021C35087).

## Conflict of Interest

The authors declare that the research was conducted in the absence of any commercial or financial relationships that could be construed as a potential conflict of interest.

## Publisher's Note

All claims expressed in this article are solely those of the authors and do not necessarily represent those of their affiliated organizations, or those of the publisher, the editors and the reviewers. Any product that may be evaluated in this article, or claim that may be made by its manufacturer, is not guaranteed or endorsed by the publisher.
